# Discontinuous color variation in the assassin bug species *Tiarodes miyamotoi* (Hemiptera: Reduviidae: Reduviinae)

**DOI:** 10.3897/BDJ.4.e10225

**Published:** 2016-10-19

**Authors:** Tadashi Ishikawa

**Affiliations:** ‡ Laboratory of Entomology, Faculty of Agriculture, Tokyo University of Agriculture, Atsugi-shi, Kanagawa, Japan

**Keywords:** Heteroptera, intraspecific variation, Japan, Reduviidae, Reduviinae, Ryukyu Islands, *Tiarodes miyamotoi*

## Abstract

**Background:**

The reduviine assassin bug, *Tiarodes miyamotoi* Ishikawa, Cai and Tomokuni, 2005 (Hemiptera: Heteroptera: Reduviidae: Reduviinae), has so far been known only from the Yaeyama Islands of Japan and no major intraspecific variation has been reported in this species.

**New information:**

This is the first record of this species from Okinawa-honto Island as the northernmost locality. Furthermore, an apparently discontinuous intraspecific variation in coloration has been confirmed between populations from the Yaeyama and Okinawa-honto Islands; this variation has been reported for the first time in the genus *Tiarodes* Burmeister, 1835, which comprises approximately 90 species.

## Introduction

The reduviine assassin bug, *Tiarodes miyamotoi* Ishikawa, Cai and Tomokuni, 2005, was described from Ishigaki-jima and Iriomote-jima Islands of the Yaeyama Islands, located in the southwestern Ryukyu Islands, Japan (Ishikawa et al. 2005). In the original description of the species, these authors examined a total of 22 specimens (12 males and 10 females) collected from nine localities on the two islands, and noted intraspecific variation among individuals such as body and appendage length and between sexes such as eye size, antennal length, and pronotal lobe length; these differences are relatively small, being universally found in species belonging to the genus *Tiarodes* Burmeister, 1835 and its relatives (Ishikawa et al. 2005). Since then, no additional information on the distribution range and intraspecific variation of this species has been published (Ishikawa and Miyamoto 2012, Ishikawa 2016).

Recently, I examined three specimens of *Tiarodes* obtained from Okinawa-honto Island, central Ryukyu Islands. These specimens looked relatively different at first glance from *T. miyamotoi* owing to a wholly darkened body and different color pattern. However, a detailed morphological examination, including genital structures, revealed that the three specimens corresponded exactly to *T. miyamotoi*, even though coloration was inconsistent between populations from the Yaeyama and Okinawa-honto Islands.

I herein record *T. miyamotoi* from Okinawa-honto Island as a new distribution record and the northernmost locality for the first time, and describe the difference in coloration between the populations as a remarkable intraspecific variation of *T. miyamotoi*. In addition, I provide a brief discussion on intraspecific variations in *Tiarodes* and indicate the taxonomic significance.

## Materials and methods

**Figure 1.**
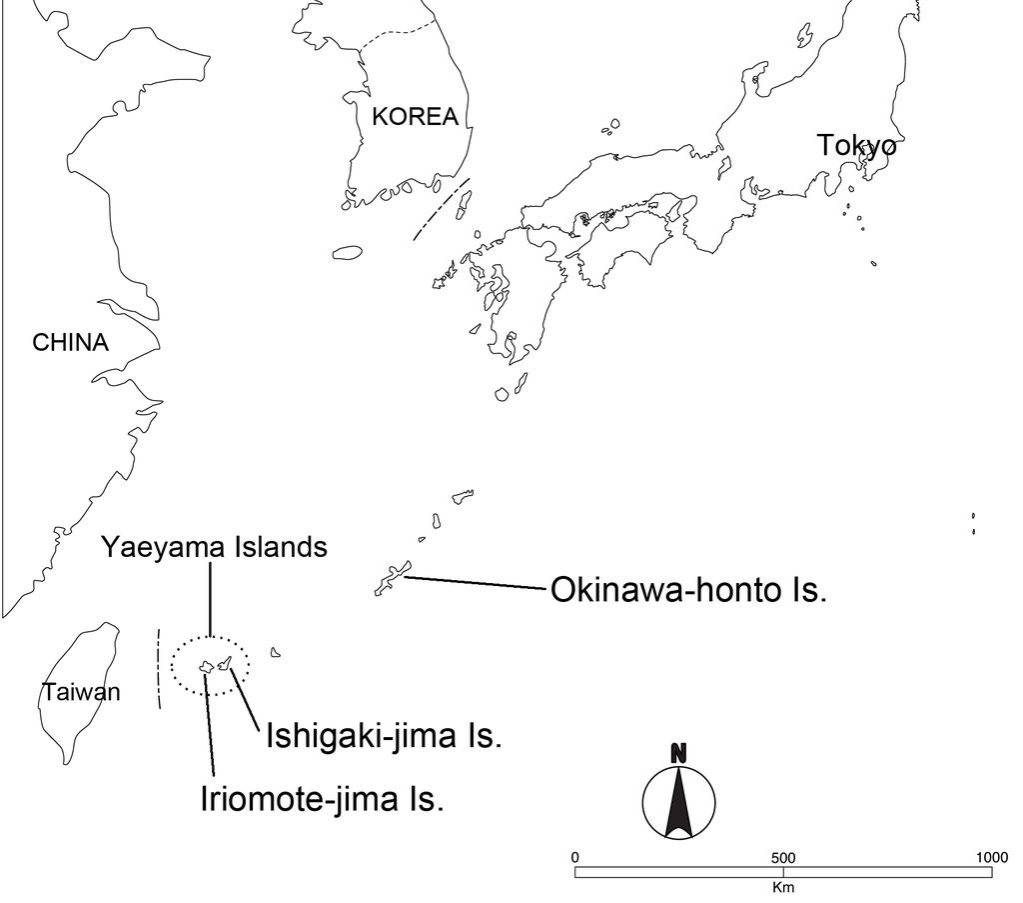
Locations of Okinawa-honto Island, Ishigaki-jima Island, and Iriomote-jima Island.

A total of 15 dried specimens were used, of which 12 specimens were collected from the Yaeyama Islands (Ishigaki-jima and Iriomote-jima Islands) and 3 from Okinawa-honto Island (Fig. 1). Morphological observations were made using an Olympus SZ61 binocular microscope. Digital images of the specimens were taken with a Nikon D200 digital camera body and Nikon AF Micro Nikkor 60mm f/2.8D lens, and with a Keyence digital microscope system (VHX-1000 digital microscope, VHX-S50 observation system, VHX-1100 multi scan, and VH-Z20R zoom lens).

The terminology used herein generally follows that of Davis (1966). The apparent labial segments I, II, and III are here referred to as rostral segments I, II, and III; these apparent labial (rostral) segments I, II, and III correspond to the “true” labial segments II, III, and IV, respectively.

All materials including type specimens have been preserved in the Insect Collection (IC) at the Laboratory of Entomology, Tokyo University of Agriculture, Japan (ELTUA).

## Taxon treatments

### Tiarodes miyamotoi

Ishikawa, Ca﻿i and Tomokuni, 2005

Tiarodes miyamotoi Ishikawa, Cai and Tomokuni, 2005, p. 282–285, orig. descr. and figs; Ishikawa and Miyamoto, 2012, p. 269, descr. and figs; Ishikawa, 2016, p. 447, cat.

#### Materials

- **Type status:** Paratype. **Occurrence:** catalogNumber: 2016-00004; recordedBy: Tadafumi Nakata; individualCount: 1; sex: female; lifeStage: adult; **Taxon:** scientificName: *Tiarodes miyamotoi* Ishikawa, Cai and Tomokuni, 2005; namePublishedIn: 2005; kingdom: Animalia; phylum: Arthropoda; class: Insecta; order: Hemiptera; family: Reduviidae; genus: Tiarodes; specificEpithet: miyamotoi; scientificNameAuthorship: Ishikawa, Cai and Tomokuni; **Location:** islandGroup: Ryukyu Islands; island: Ishigaki-jima Island; country: Japan; stateProvince: Okinawa; municipality: Ishigaki City; locality: Mt. Yarabu-dake; decimalLatitude: 24.4401; decimalLongitude: 124.0869; geodeticDatum: WGS84; **Identification:** identifiedBy: Tadashi Ishikawa; dateIdentified: 2005; **Event:** samplingProtocol: none specified; eventDate: 2000-07-11; **Record Level:** institutionCode: ELTUA; collectionCode: IC; basisOfRecord: PreservedSpecimen
- **Type status:** Paratype. **Occurrence:** catalogNumber: 2016-00005; recordedBy: Tadafumi Nakata; individualCount: 1; sex: female; lifeStage: adult; **Taxon:** scientificName: *Tiarodes miyamotoi* Ishikawa, Cai and Tomokuni, 2005; namePublishedIn: 2005; kingdom: Animalia; phylum: Arthropoda; class: Insecta; order: Hemiptera; family: Reduviidae; genus: Tiarodes; specificEpithet: miyamotoi; scientificNameAuthorship: Ishikawa, Cai and Tomokuni; **Location:** islandGroup: Ryukyu Islands; island: Ishigaki-jima Island; country: Japan; stateProvince: Okinawa; municipality: Ishigaki City; locality: Mt. Yarabu-dake; decimalLatitude: 24.4401; decimalLongitude: 124.0869; geodeticDatum: WGS84; **Identification:** identifiedBy: Tadashi Ishikawa; dateIdentified: 2005; **Event:** samplingProtocol: none specified; eventDate: 2000-07-11; **Record Level:** institutionCode: ELTUA; collectionCode: IC; basisOfRecord: PreservedSpecimen
- **Type status:** Paratype. **Occurrence:** catalogNumber: 2016-00006; recordedBy: Tadafumi Nakata; individualCount: 1; sex: female; lifeStage: adult; **Taxon:** scientificName: *Tiarodes miyamotoi* Ishikawa, Cai and Tomokuni, 2005; namePublishedIn: 2005; kingdom: Animalia; phylum: Arthropoda; class: Insecta; order: Hemiptera; family: Reduviidae; genus: Tiarodes; specificEpithet: miyamotoi; scientificNameAuthorship: Ishikawa, Cai and Tomokuni; **Location:** islandGroup: Ryukyu Islands; island: Ishigaki-jima Island; country: Japan; stateProvince: Okinawa; municipality: Ishigaki City; locality: Mt. Yarabu-dake; decimalLatitude: 24.4401; decimalLongitude: 124.0869; geodeticDatum: WGS84; **Identification:** identifiedBy: Tadashi Ishikawa; dateIdentified: 2005; **Event:** samplingProtocol: none specified; eventDate: 2000-07-19; **Record Level:** institutionCode: ELTUA; collectionCode: IC; basisOfRecord: PreservedSpecimen
- **Type status:** Paratype. **Occurrence:** catalogNumber: 2016-00007; recordedBy: Tadafumi Nakata; individualCount: 1; sex: female; lifeStage: adult; **Taxon:** scientificName: *Tiarodes miyamotoi* Ishikawa, Cai and Tomokuni, 2005; namePublishedIn: 2005; kingdom: Animalia; phylum: Arthropoda; class: Insecta; order: Hemiptera; family: Reduviidae; genus: Tiarodes; specificEpithet: miyamotoi; scientificNameAuthorship: Ishikawa, Cai and Tomokuni; **Location:** islandGroup: Ryukyu Islands; island: Ishigaki-jima Island; country: Japan; stateProvince: Okinawa; municipality: Ishigaki City; locality: Mt. Yarabu-dake; decimalLatitude: 24.4401; decimalLongitude: 124.0869; geodeticDatum: WGS84; **Identification:** identifiedBy: Tadashi Ishikawa; dateIdentified: 2005; **Event:** samplingProtocol: none specified; eventDate: 2000-07-19; **Record Level:** institutionCode: ELTUA; collectionCode: IC; basisOfRecord: PreservedSpecimen
- **Type status:** Paratype. **Occurrence:** catalogNumber: 2016-00008; recordedBy: Tadafumi Nakata; individualCount: 1; sex: female; lifeStage: adult; **Taxon:** scientificName: *Tiarodes miyamotoi* Ishikawa, Cai and Tomokuni, 2005; namePublishedIn: 2005; kingdom: Animalia; phylum: Arthropoda; class: Insecta; order: Hemiptera; family: Reduviidae; genus: Tiarodes; specificEpithet: miyamotoi; scientificNameAuthorship: Ishikawa, Cai and Tomokuni; **Location:** islandGroup: Ryukyu Islands; island: Ishigaki-jima Island; country: Japan; stateProvince: Okinawa; municipality: Ishigaki City; locality: Mt. Yarabu-dake; decimalLatitude: 24.4401; decimalLongitude: 124.0869; geodeticDatum: WGS84; **Identification:** identifiedBy: Tadashi Ishikawa; dateIdentified: 2005; **Event:** samplingProtocol: none specified; eventDate: 2000-07-19; **Record Level:** institutionCode: ELTUA; collectionCode: IC; basisOfRecord: PreservedSpecimen
- **Type status:** Paratype. **Occurrence:** catalogNumber: 2016-00009; recordedBy: Tadafumi Nakata; individualCount: 1; sex: female; lifeStage: adult; **Taxon:** scientificName: *Tiarodes miyamotoi* Ishikawa, Cai and Tomokuni, 2005; namePublishedIn: 2005; kingdom: Animalia; phylum: Arthropoda; class: Insecta; order: Hemiptera; family: Reduviidae; genus: Tiarodes; specificEpithet: miyamotoi; scientificNameAuthorship: Ishikawa, Cai and Tomokuni; **Location:** islandGroup: Ryukyu Islands; island: Ishigaki-jima Island; country: Japan; stateProvince: Okinawa; municipality: Ishigaki City; locality: Mt. Yarabu-dake; decimalLatitude: 24.4401; decimalLongitude: 124.0869; geodeticDatum: WGS84; **Identification:** identifiedBy: Tadashi Ishikawa; dateIdentified: 2005; **Event:** samplingProtocol: none specified; eventDate: 2000-07-19; **Record Level:** institutionCode: ELTUA; collectionCode: IC; basisOfRecord: PreservedSpecimen
- **Type status:** Paratype. **Occurrence:** catalogNumber: 2016-00010; recordedBy: Tadafumi Nakata; individualCount: 1; sex: female; lifeStage: adult; **Taxon:** scientificName: *Tiarodes miyamotoi* Ishikawa, Cai and Tomokuni, 2005; namePublishedIn: 2005; kingdom: Animalia; phylum: Arthropoda; class: Insecta; order: Hemiptera; family: Reduviidae; genus: Tiarodes; specificEpithet: miyamotoi; scientificNameAuthorship: Ishikawa, Cai and Tomokuni; **Location:** islandGroup: Ryukyu Islands; island: Ishigaki-jima Island; country: Japan; stateProvince: Okinawa; municipality: Ishigaki City; locality: Mt. Yarabu-dake; decimalLatitude: 24.4401; decimalLongitude: 124.0869; geodeticDatum: WGS84; **Identification:** identifiedBy: Tadashi Ishikawa; dateIdentified: 2005; **Event:** samplingProtocol: none specified; eventDate: 2000-07-19; **Record Level:** institutionCode: ELTUA; collectionCode: IC; basisOfRecord: PreservedSpecimen
- **Type status:** Paratype. **Occurrence:** catalogNumber: 2016-00011; recordedBy: Tomoyuki Tsuru; individualCount: 1; sex: male; lifeStage: adult; **Taxon:** scientificName: *Tiarodes miyamotoi* Ishikawa, Cai and Tomokuni, 2005; namePublishedIn: 2005; kingdom: Animalia; phylum: Arthropoda; class: Insecta; order: Hemiptera; family: Reduviidae; genus: Tiarodes; specificEpithet: miyamotoi; scientificNameAuthorship: Ishikawa, Cai and Tomokuni; **Location:** islandGroup: Ryukyu Islands; island: Iriomote-jima Island; country: Japan; stateProvince: Okinawa; municipality: Taketomi Town; locality: Shirahama-rindo; decimalLatitude: 24.3671; decimalLongitude: 123.7599; geodeticDatum: WGS84; **Identification:** identifiedBy: Tadashi Ishikawa; dateIdentified: 2005; **Event:** samplingProtocol: none specified; eventDate: 2002-06-12; **Record Level:** institutionCode: ELTUA; collectionCode: IC; basisOfRecord: PreservedSpecimen
- **Type status:** Paratype. **Occurrence:** catalogNumber: 2016-00012; recordedBy: Seidai Nagashima; individualCount: 1; sex: male; lifeStage: adult; **Taxon:** scientificName: *Tiarodes miyamotoi* Ishikawa, Cai and Tomokuni, 2005; namePublishedIn: 2005; kingdom: Animalia; phylum: Arthropoda; class: Insecta; order: Hemiptera; family: Reduviidae; genus: Tiarodes; specificEpithet: miyamotoi; scientificNameAuthorship: Ishikawa, Cai and Tomokuni; **Location:** islandGroup: Ryukyu Islands; island: Iriomote-jima Island; country: Japan; stateProvince: Okinawa; municipality: Taketomi Town; locality: Shirahama-rindo; decimalLatitude: 24.3671; decimalLongitude: 123.7599; geodeticDatum: WGS84; **Identification:** identifiedBy: Tadashi Ishikawa; dateIdentified: 2005; **Event:** samplingProtocol: none specified; eventDate: 2003-05-30; **Record Level:** institutionCode: ELTUA; collectionCode: IC; basisOfRecord: PreservedSpecimen
- **Type status:** Paratype. **Occurrence:** catalogNumber: 2016-00013; recordedBy: Seidai Nagashima; individualCount: 1; sex: male; lifeStage: adult; **Taxon:** scientificName: *Tiarodes miyamotoi* Ishikawa, Cai and Tomokuni, 2005; namePublishedIn: 2005; kingdom: Animalia; phylum: Arthropoda; class: Insecta; order: Hemiptera; family: Reduviidae; genus: Tiarodes; specificEpithet: miyamotoi; scientificNameAuthorship: Ishikawa, Cai and Tomokuni; **Location:** islandGroup: Ryukyu Islands; island: Iriomote-jima Island; country: Japan; stateProvince: Okinawa; municipality: Taketomi Town; locality: Shirahama; decimalLatitude: 24.363; decimalLongitude: 123.7546; geodeticDatum: WGS84; **Identification:** identifiedBy: Tadashi Ishikawa; dateIdentified: 2005; **Event:** samplingProtocol: none specified; eventDate: 2003-05-31; **Record Level:** institutionCode: ELTUA; collectionCode: IC; basisOfRecord: PreservedSpecimen
- **Type status:** Paratype. **Occurrence:** catalogNumber: 2016-00014; recordedBy: Tomoyuki Tsuru; individualCount: 1; sex: male; lifeStage: adult; **Taxon:** scientificName: *Tiarodes miyamotoi* Ishikawa, Cai and Tomokuni, 2005; namePublishedIn: 2005; kingdom: Animalia; phylum: Arthropoda; class: Insecta; order: Hemiptera; family: Reduviidae; genus: Tiarodes; specificEpithet: miyamotoi; scientificNameAuthorship: Ishikawa, Cai and Tomokuni; **Location:** islandGroup: Ryukyu Islands; island: Iriomote-jima Island; country: Japan; stateProvince: Okinawa; municipality: Taketomi Town; locality: Sonai; decimalLatitude: 24.3856; decimalLongitude: 123.75; geodeticDatum: WGS84; **Identification:** identifiedBy: Tadashi Ishikawa; dateIdentified: 2005; **Event:** samplingProtocol: none specified; eventDate: 2003-05-27; **Record Level:** institutionCode: ELTUA; collectionCode: IC; basisOfRecord: PreservedSpecimen
- **Type status:** Other material. **Occurrence:** catalogNumber: 2016-00015; recordedBy: K. Kurihara; individualCount: 1; sex: male; lifeStage: adult; **Taxon:** scientificName: *Tiarodes miyamotoi* Ishikawa, Cai and Tomokuni, 2005; namePublishedIn: 2005; kingdom: Animalia; phylum: Arthropoda; class: Insecta; order: Hemiptera; family: Reduviidae; genus: Tiarodes; specificEpithet: miyamotoi; scientificNameAuthorship: Ishikawa, Cai and Tomokuni; **Location:** islandGroup: Ryukyu Islands; island: Iriomote-jima Island; country: Japan; stateProvince: Okinawa; municipality: Taketomi Town; locality: Mt. Sonai-dake; decimalLatitude: 24.3857; decimalLongitude: 123.7523; geodeticDatum: WGS84; **Identification:** identifiedBy: Tadashi Ishikawa; dateIdentified: 2016; **Event:** samplingProtocol: none specified; eventDate: 2002-06-18; **Record Level:** institutionCode: ELTUA; collectionCode: IC; basisOfRecord: PreservedSpecimen
- **Type status:** Other material. **Occurrence:** catalogNumber: 2016-00001; recordedBy: Kuki Baba; individualCount: 1; sex: male; lifeStage: adult; **Taxon:** scientificName: *Tiarodes miyamotoi* Ishikawa, Cai and Tomokuni, 2005; namePublishedIn: 2005; kingdom: Animalia; phylum: Arthropoda; class: Insecta; order: Hemiptera; family: Reduviidae; genus: Tiarodes; specificEpithet: miyamotoi; scientificNameAuthorship: Ishikawa, Cai and Tomokuni; **Location:** islandGroup: Ryukyu Islands; island: Okinawa-honto Island; country: Japan; stateProvince: Okinawa; municipality: Ogimi Village; locality: Kijoka; decimalLatitude: 26.7043; decimalLongitude: 128.1478; geodeticDatum: WGS84; **Identification:** identifiedBy: Tadashi Ishikawa; dateIdentified: 2016; **Event:** samplingProtocol: none specified; eventDate: 2013-06-17; **Record Level:** institutionCode: ELTUA; collectionCode: IC; basisOfRecord: PreservedSpecimen
- **Type status:** Other material. **Occurrence:** catalogNumber: 2016-00002; recordedBy: Takeru Naka; individualCount: 1; sex: male; lifeStage: adult; **Taxon:** scientificName: *Tiarodes miyamotoi* Ishikawa, Cai and Tomokuni, 2005; namePublishedIn: 2005; kingdom: Animalia; phylum: Arthropoda; class: Insecta; order: Hemiptera; family: Reduviidae; genus: Tiarodes; specificEpithet: miyamotoi; scientificNameAuthorship: Ishikawa, Cai and Tomokuni; **Location:** islandGroup: Ryukyu Islands; island: Okinawa-honto Island; country: Japan; stateProvince: Okinawa; municipality: Ogimi Village; locality: Ogimi; decimalLatitude: 26.684; decimalLongitude: 128.137; geodeticDatum: WGS84; **Identification:** identifiedBy: Tadashi Ishikawa; dateIdentified: 2016; **Event:** samplingProtocol: none specified; eventDate: 2013-07-01; **Record Level:** institutionCode: ELTUA; collectionCode: IC; basisOfRecord: PreservedSpecimen
- **Type status:** Other material. **Occurrence:** catalogNumber: 2016-00003; recordedBy: Takeru Naka; individualCount: 1; sex: male; lifeStage: adult; **Taxon:** scientificName: *Tiarodes miyamotoi* Ishikawa, Cai and Tomokuni, 2005; namePublishedIn: 2005; kingdom: Animalia; phylum: Arthropoda; class: Insecta; order: Hemiptera; family: Reduviidae; genus: Tiarodes; specificEpithet: miyamotoi; scientificNameAuthorship: Ishikawa, Cai and Tomokuni; **Location:** islandGroup: Ryukyu Islands; island: Okinawa-honto Island; country: Japan; stateProvince: Okinawa; municipality: Ogimi Village; locality: Ogimi; decimalLatitude: 26.684; decimalLongitude: 128.137; geodeticDatum: WGS84; **Identification:** identifiedBy: Tadashi Ishikawa; dateIdentified: 2016; **Event:** samplingProtocol: none specified; eventDate: 2013-07-01; **Record Level:** institutionCode: ELTUA; collectionCode: IC; basisOfRecord: PreservedSpecimen

#### Intraspecific variation

In this species, a discontinuous geographical variation occurs between the Yaeyama and Okinawa-honto Island populations, the two forms are tentatively called “type-Y” and “type-O”, respectively. These types differ distinctly in coloration, but not in external morphology, including genital structures. The differences between the types are as follows (also see Table 1):

Head red, with irregular black markings in type-Y (Figs 2a, 3a), and blackish, more or less suffused with red on ventral disc, on dorsum of anterior lobe, and on both sides of posterior lobe in type-O (Figs 2c, 3b). Rostral segments I and II reddish with yellowish suffusion in type-Y (Figs 2b, 3a), and blackish in type-O (Figs 2d, 3b). Pronotum black on collar, red on anterior lobe, and dark red on posterior lobe in type-Y (Fig. 2a), and wholly blackish, with reddish suffusion on anterior disc of anterior lobe in type-O (Fig. 2c). Coxae and trochanters red with dark markings in type-Y (Figs 2b, 4a), and black with irregular red to yellow markings in type-O (Figs 2c, 4b). Femora each black on extreme apex in type-Y (Figs 2b, 4a), and black on apical fifth in type-O (Figs 2d, 4b). Large yellow spot of hemelytral corium vivid in type-Y (Fig. 2a), and obscure in type-O (Fig. 2c). Abdominal sternites III to VII reddish, darkened along anterior margin in type-Y (Fig. 2b), and entirely reddish in type-O (Fig. 2d). Abdominal laterotergites III to VI entirely red in type-Y (Fig. 2a, b), and red with anterior corner of each segment black in type-O (Fig. 2c, d). Abdominal laterotergite VII red in type-Y (Figs 2a, b, 5a), and black in type-O (Figs 2c, d, 5b). Male genital segments yellowish with irregular obscure markings in type-Y (Figs 2b, 5a), and entirely black in type-O (Figs 2d, 5b).

#### Improved diagnosis

Ishikawa et al. (2005) provided a diagnosis of *T. miyamotoi* in its original description. However, the intraspecific variation confirmed in this study makes the diagnosis revised. Therefore, this species is re-diagnosed as follow:

*Tiarodes miyamotoi* is recognized by the following combination of characters: body 15–20 mm long; head a little longer than pronotum; pronotal collar well developed and distinctly projecting on both sides; humeral width 1.2 times as long as pronotum in the midline; mesosternum without median sulcus; meso- and metasterna without median carina; hemelytral corium with one large, vivid to obscure yellow spot; venter of abdomen mostly generally reddish; abdominal laterotergite II blackish; parameres longitudinally carinate ventrally in apical half.

#### Distribution

Japan: the Ryukyu Islands [Okinawa-honto Island, the Yaeyama Islands (Ishigaki-jima Island and Iriomote-jima Island)]. This species was first recorded from Okinawa-honto Island.

## Discussion

As described above, *T. miyamotoi* shows a remarkable intraspecific variation in coloration, although its external morphology is insignificantly variable among the specimens obtained throughout its distribution range. Interestingly, the variation is discontinuous and clearly geographically separated between the Yaeyama and Okinawa-honto Islands (Fig. 1). As the two populations occurring in these two island groups are different in coloration, with the sea lying between the two areas as a physical barrier, it is possible that each of the populations could be recognized as a subspecies; however, we postpone any final conclusion about the subspecies until more specimens of this species, especially those from Okinawa-honto Island, are studied further.

Major differences in coloration within the species have been recognized in other congeners of *Tiarodes*. Cai et al. (2001) documented various color patterns of abdominal venter in *T. venenatus* Cai and Sun in Cai et al., 2001 and *T. pictus* Cai and Tomokuni in Cai et al., 2001. The former was described from mainland China (Zhejiang, Fujian) and Taiwan based on five specimens, and the latter from Taiwan based on seven specimens (Cai et al. 2001). However, based on their descriptions and illustrations, the variability appears to be continuous in each species with no relation to geographical tendency or gap. Therefore, this is the only example of discontinuous variation documented in the genus *Tiarodes* so far.

In *Tiarodes*, approximately 90 species have been described (Miller 1959, Cai et al. 2001, Tomokuni and Cai 2002, Ishikawa et al. 2005, Ishikawa and Miyamoto 2012). Most species have been described based on a single or few specimens owing to their limited abundance, and were distinguished from each other mainly by differences in coloration. Miller (1959) provided comprehensive identification keys for more than 80 species, using primarily coloration characteristics. Since the presence of remarkable intraspecific variations in coloration is evident as mentioned above, it is possible that two or more populations actually belonging to one species have been described as different species in the congeners of *Tiarodes*. Therefore, it is necessary to accurately evaluate the color characteristics defining a species of *Tiarodes* to revise the taxonomy and descriptions. In addition, it is also important to detect informative characters from the external morphology and to analyze molecular data to effectively discriminate between *Tiarodes* species.

## Supplementary Material

XML Treatment for Tiarodes miyamotoi

## Figures and Tables

**Figure 2a.**
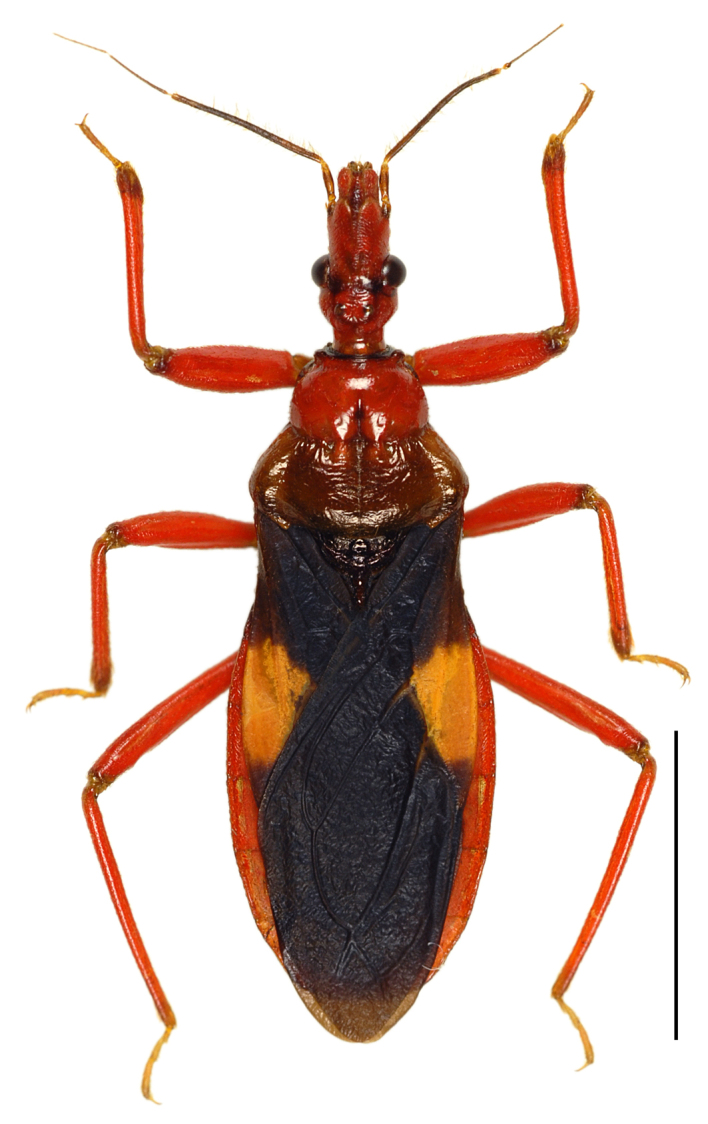
*Tiarodes miyamotoi* Ishikawa, Cai and Tomokuni, 2005, dorsal, Iriomote-jima Is. (LETUA_IC 2016-00015)

**Figure 2b.**
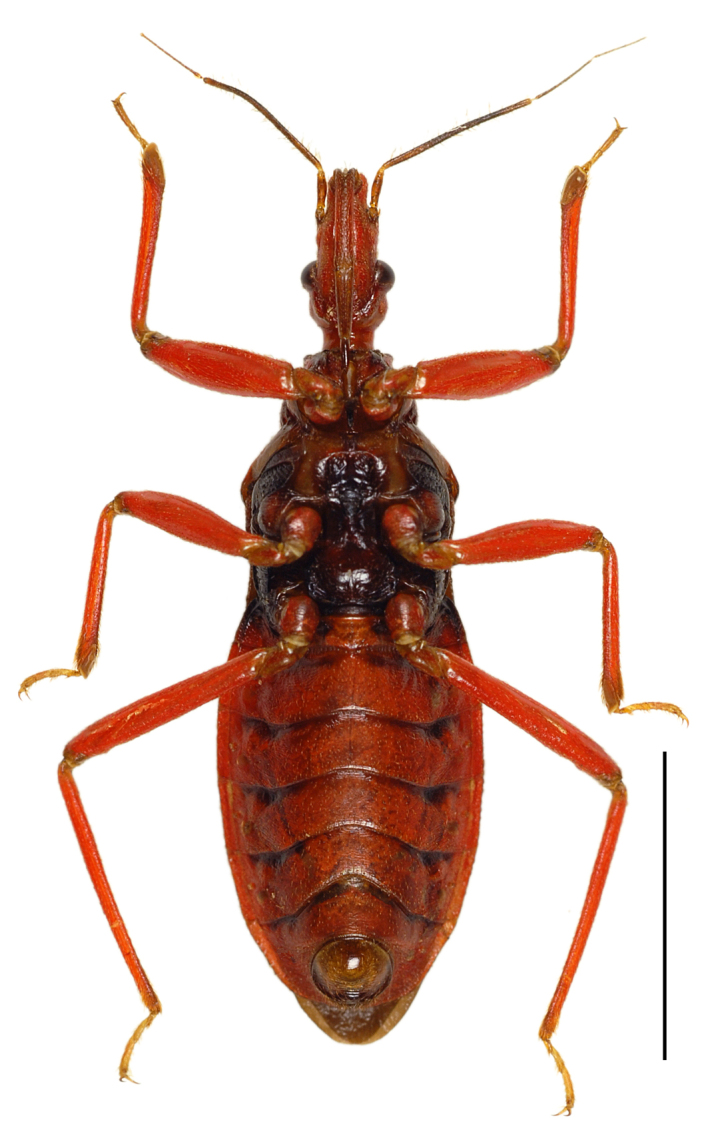
*Tiarodes miyamotoi* Ishikawa, Cai and Tomokuni, 2005, ventral, Iriomote-jima Is. (LETUA_IC 2016-00015)

**Figure 2c.**
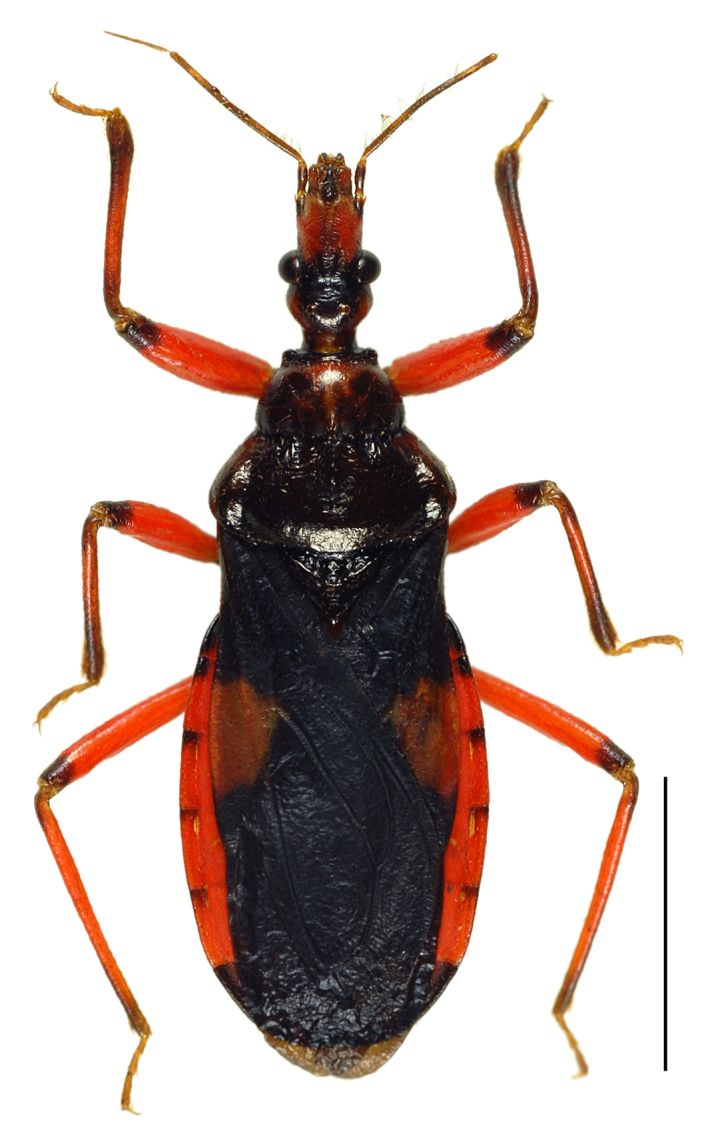
*Tiarodes miyamotoi* Ishikawa, Cai and Tomokuni, 2005, dorsal, Okinawa-honto Is. (LETUA_IC 2016-00001)

**Figure 2d.**
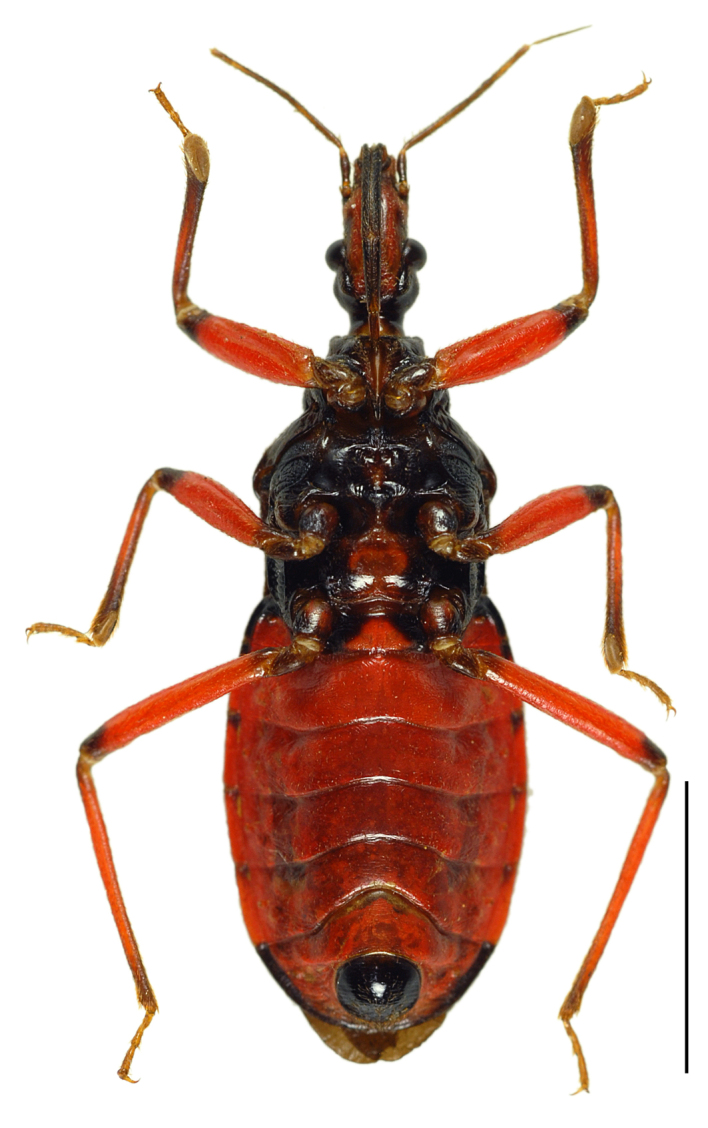
*Tiarodes miyamotoi* Ishikawa, Cai and Tomokuni, 2005, ventral, Okinawa-honto Is. (LETUA_IC 2016-00001)

**Figure 3a.**
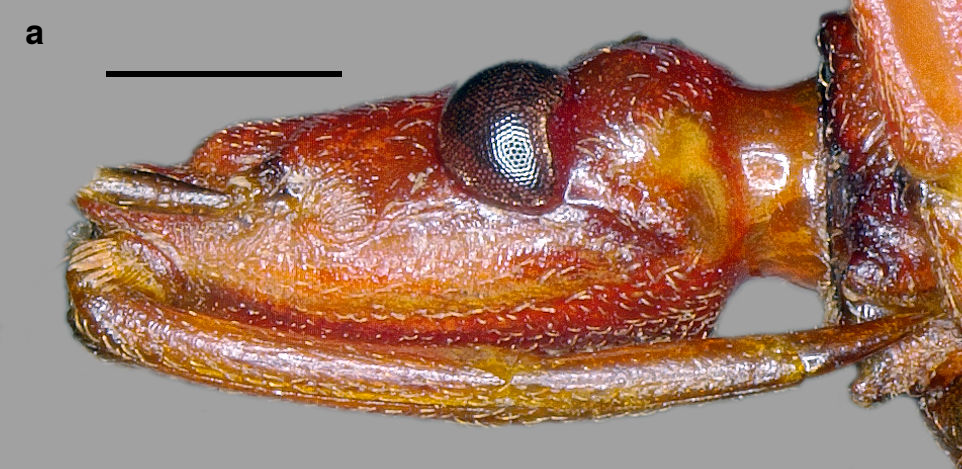
*Tiarodes miyamotoi* Ishikawa, Cai and Tomokuni, 2005, Iriomote-jima Is. (LETUA_IC 2016-00015)

**Figure 3b.**
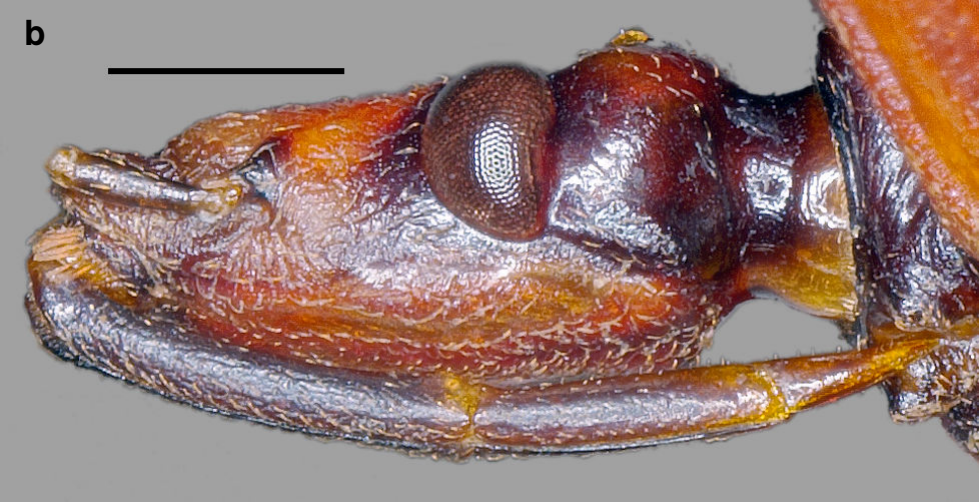
*Tiarodes miyamotoi* Ishikawa, Cai and Tomokuni, 2005, Okinawa-honto Is. (LETUA_IC 2016-00001)

**Figure 4a.**
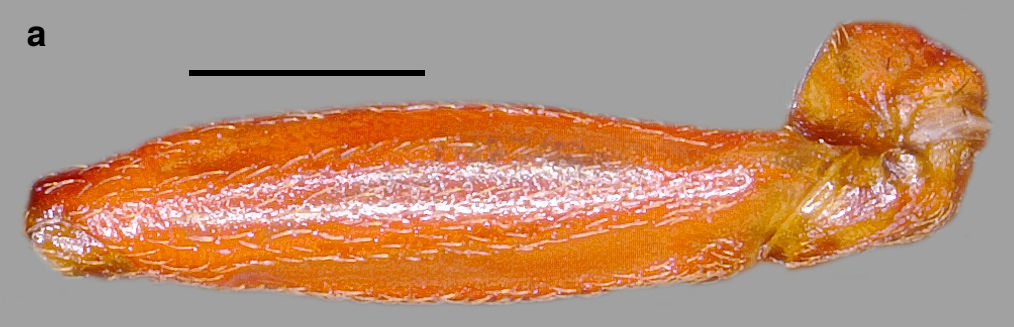
*Tiarodes miyamotoi* Ishikawa, Cai and Tomokuni, 2005, Iriomote-jima Is. (LETUA_IC 2016-00015)

**Figure 4b.**
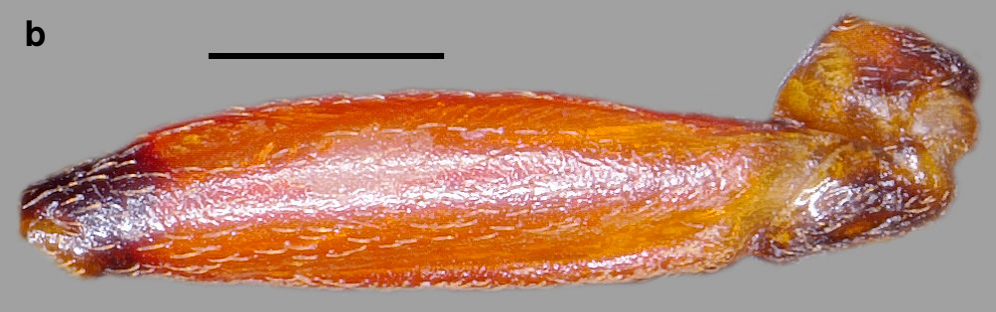
*Tiarodes miyamotoi* Ishikawa, Cai and Tomokuni, 2005, Okinawa-honto Is. (LETUA_IC 2016-00001)

**Figure 5a.**
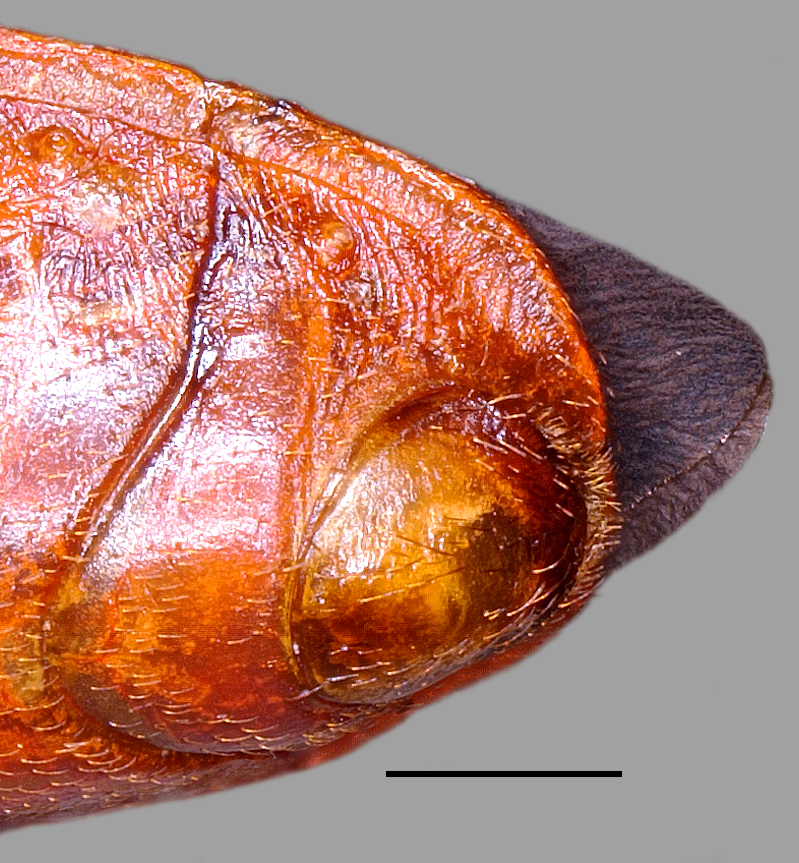
*Tiarodes miyamotoi* Ishikawa, Cai and Tomokuni, 2005, Iriomote-jima Is. (LETUA_IC 2016-00015)

**Figure 5b.**
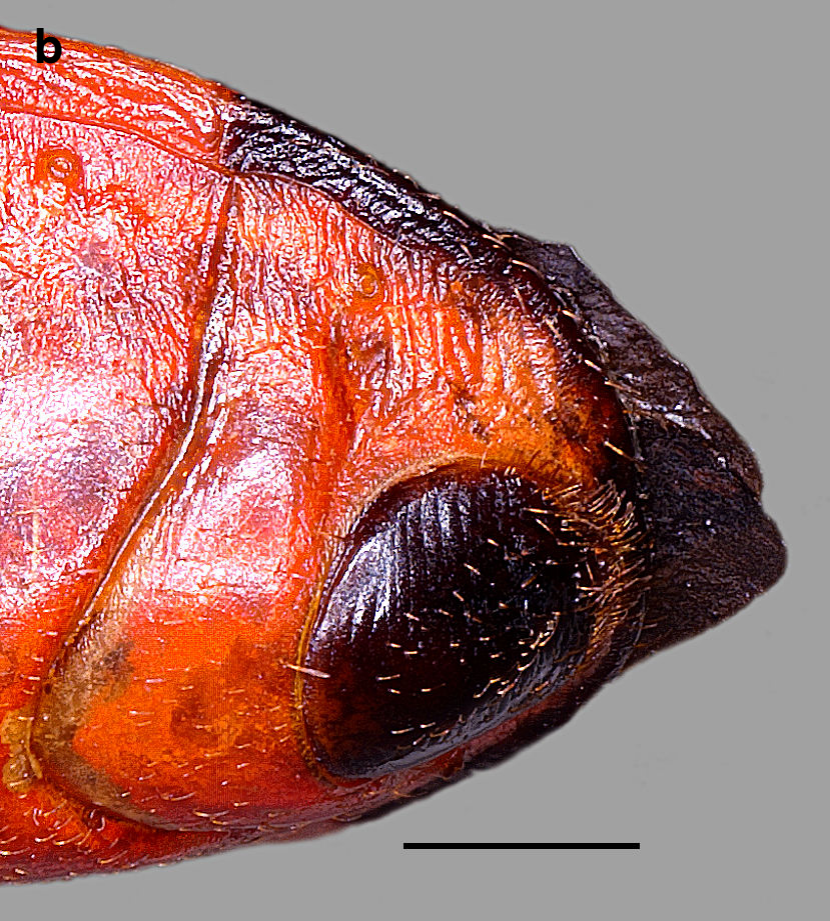
*Tiarodes miyamotoi* Ishikawa, Cai and Tomokuni, 2005, Okinawa-honto Is. (LETUA_IC 2016-00001)

**Table 1.** Differential diagnosis of type-Y. and type-O of *Tiarodes miyamotoi* Ishikawa, Cai and Tomokuni, 2005

	type-Y	type-O
Head	red, with irregular black markings	blackish, more or less suffused with red on ventral disc, on dorsum of anterior lobe, and on both sides of posterior lobe
Rostral segments I and II	reddish with yellowish suffusion	blackish
Pronotum	black on collar, red on anterior lobe, dark red on posterior lobe	wholly blackish, with reddish suffusion on anterior disc of anterior lobe
Coxae and trochanters	red with dark markings	black with irregular red to yellow markings
Femora	black on extreme apex	black on apical fifth
Large yellow spot of hemelytral corium	vivid	obscure
Abdominal sternites III to VII	reddish, darkened along anterior margin	entirely reddish
Abdominal laterotergites III to VI	entirely red	red with anterior corner of each segment black
Abdominal laterotergite VII	red	black
Male genital segments	yellowish with irregular obscure markings	entirely black
